# Critical gene network and signaling pathway analysis of the extracellular signal-regulated kinase (ERK) pathway in ischemic stroke

**DOI:** 10.3389/fnmol.2025.1604670

**Published:** 2025-06-25

**Authors:** Rui Mao, Lei Wang, Haitao Zhang, Jiaojiao Gong, Hua Liu

**Affiliations:** Department of Neurology, The Third People’s Hospital of Chengdu, Chengdu, Sichuan, China

**Keywords:** ischemic stroke, ERK pathway, GADD45, DUSP1, machine learning, nomogram

## Abstract

**Background and objective:**

Ischemic stroke remains a leading cause of morbidity worldwide, demanding reliable biomarkers and mechanistic insights to inform personalized diagnostic and therapeutic strategies. We sought to integrate multiple ischemic stroke transcriptomic datasets, identify key extracellular signal-regulated kinase (ERK) pathway–related biomarkers, delineate immune–stromal heterogeneity, and develop a nomogram for clinical risk assessment.

**Methods:**

We retrieved three public microarray datasets (GSE22255, GSE16561, GSE58294) and merged two of them (GSE22255, GSE16561) into a discovery cohort after stringent batch correction. Differential expression analyses were performed using the limma package in R, followed by weighted gene co-expression network analysis (WGCNA) to identify ERK-associated gene modules. Gene Ontology (GO) enrichment and protein–protein interaction (PPI) network analyses further elucidated the functional and interaction landscapes of the key ERK pathway genes, collectively termed GSERK. Subsequently, hub genes were prioritized using cytoHubba, and their diagnostic utility was validated by receiver operating characteristic (ROC) analyses in both discovery and validation cohorts. Four machine learning algorithms (Boruta, SVM, LASSO, random forest) corroborated hub gene robustness. Finally, we stratified ischemic stroke samples by immune–stromal profiling and constructed a GSERK-based nomogram to predict stroke risk.

**Results:**

A total of 140 differentially expressed genes (DEGs) were identified, with the ERK-related subset (GSERK) highlighted for its pivotal roles in ischemic stroke pathogenesis. Five hub GSERK genes (GADD45A, DUSP1, IL1B, JUN, and GADD45B) emerged from cytoHubba. DUSP1, GADD45A, and GADD45B showed robust diagnostic accuracy (AUC: 0.75–0.91), confirmed across discovery and validation sets. Immune–stromal clustering revealed two distinct stroke subgroups with hyperinflammatory or quiescent stromal phenotypes. A GSERK-based nomogram demonstrated a strong bootstrap-validated C-index, underscoring its potential for clinical risk stratification.

**Conclusion:**

These findings affirm the significance of ERK signaling in ischemic stroke, unveil critical GSERK biomarkers with promising diagnostic and therapeutic implications, and present a novel GSERK-based nomogram for precision risk assessment. Further studies, including experimental validation and multi-center clinical trials, are warranted to refine this integrative approach toward personalized stroke care.

## 1 Introduction

Ischemic stroke, a leading cause of morbidity and mortality globally, is characterized by acute interruption of cerebral blood flow. This results in a cascade of pathological events including excitotoxicity, oxidative stress, inflammation, neuronal apoptosis, and blood–brain barrier breakdown, all contributing to irreversible neurological damage and poor functional outcomes (Fanning et al., 2024; Lanas and Seron, 2021). Although reperfusion therapies such as intravenous thrombolysis and mechanical thrombectomy have revolutionized acute management, many patients remain refractory to these interventions or experience incomplete recovery, underscoring the urgent need for new therapeutic targets and reliable biomarkers for early risk stratification (Albers et al., 2018; Walter, 2022; Haupt et al., 2023).

Recent advances in transcriptomic profiling offer a promising avenue for dissecting the molecular architecture of ischemic brain injury. Yet, efforts to integrate gene expression data across independent stroke cohorts have been hampered by technical heterogeneity and batch effects, leading to inconsistencies and limited reproducibility (Candelario-Jalil et al., 2022; Kelly et al., 2021). Rigorous bioinformatics methodologies, including batch correction and weighted gene co-expression network analysis (WGCNA), have emerged as critical tools for overcoming these technical challenges, enabling precise extraction of biologically relevant pathways and molecular signatures (Hicks et al., 2018; Voß et al., 2022; Zhang and Wong, 2022).

Among the signaling cascades implicated in ischemic pathology, the extracellular signal-regulated kinase (ERK) pathway—a key arm of the mitogen-activated protein kinase (MAPK) system—has emerged as a crucial regulator of neuroinflammation, neuronal death, and vascular dysfunction (Qin et al., 2022). While prior studies have described the pathophysiological relevance of ERK signaling in ischemia, several key knowledge gaps remain: (1) the specific ERK-responsive gene networks (rather than individual genes) involved in human ischemic stroke are poorly defined; (2) there is a lack of integrative analysis across multiple patient-derived datasets to validate ERK-related signatures; and (3) the translational utility of ERK-linked biomarkers for clinical prediction or stratification remains unexplored.

In this context, we integrated multiple ischemic stroke transcriptomic datasets and applied rigorous bioinformatics approaches to identify and validate robust ERK pathway-related biomarkers (termed GSERK), define immune-stromal heterogeneity, and develop a clinically applicable nomogram for stroke risk prediction. Our findings advance the mechanistic understanding of ischemic stroke pathophysiology, laying a foundation for personalized therapeutic strategies and diagnostic precision.

## 2 Methods

### 2.1 Acquisition and download of data

Microarray datasets related to ischemic stroke were systematically retrieved from the Gene Expression Omnibus (GEO) database^1^. Three independent microarray expression datasets (GSE22255, GSE16561, and GSE58294) were selected based on stringent inclusion criteria: availability of ischemic stroke samples, matched controls, and high-quality annotation data. Specifically, dataset GSE22255 comprised 40 samples, including 20 patients with ischemic stroke and 20 controls, profiled using the Affymetrix Human Genome U133 Plus 2.0 Array (GPL570). Dataset GSE16561 contained 63 samples (39 ischemic stroke cases and 24 controls), obtained using the Illumina HumanRef-8 v3.0 Expression BeadChip (GPL6883). Dataset GSE58294 included 92 samples (69 and 23 controls), also profiled on the Affymetrix Human Genome U133 Plus 2.0 Array (GPL570).

The ERK signaling pathway genes were comprehensively extracted from the Kyoto Encyclopedia of Genes and Genomes (KEGG) pathway database (KEGG pathway ID: hsa04010^2^). The pathway encompasses a total of 300 annotated genes. These genes were systematically downloaded, and further bioinformatics analyses were conducted to explore their roles and interactions within ischemic stroke pathology.

### 2.2 Dataset curation and preprocessing

Three ischemic stroke transcriptomic datasets were retrieved from the GEO. Raw RNA expression data and platform annotation files (GPL) were obtained using the GEOquery package in R (v2.68.0). Gene symbols were annotated for each dataset separately. To ensure a robust and integrative analysis, datasets GSE22255 and GSE16561 were merged into a combined discovery cohort, while GSE58294 served as an independent validation set. Batch effects introduced by different sequencing platforms were corrected using the ComBat function from the sva package in R.

To evaluate the distribution of gene expression before and after batch effect correction, box plots of the merged dataset were generated. Additionally, uniform manifold approximation and projection (UMAP) analysis was performed using the umap package in R to visualize the overall structure of the dataset before and after batch effect removal. This analysis facilitated the assessment of clustering patterns among ischemic stroke and control samples, ensuring the integrity and comparability of the integrated dataset.

### 2.3 Analysis and visualization of differential expression genes

Following batch effect correction, a combined dataset was obtained consisting of 103 samples, including 44 healthy controls and 59 ischemic stroke samples. To facilitate downstream comparisons, all healthy controls were placed at the beginning of the dataset, followed by the diseased samples. The analysis of differential expression genes was performed using the limma package in R. Genes were considered differentially expressed if they met the following criteria: an adjusted *p*-value (Benjamini–Hochberg correction) of <0.05 and an absolute log2 fold change (|log2FC|) of >0.3. This threshold corresponds to an approximately 1.23-fold change in expression level.

A volcano plot was generated using the ggplot2 package to depict the statistical significance (−log10 of the adjusted *p*-value) against the magnitude of expression change (log2 fold change). Vertical dashed lines were drawn at −0.3 and 0.3 on the *x*-axis, and a horizontal dashed line at −log10 (0.05) on the *y*-axis, demarcating the thresholds for significance. Additionally, significantly up- and downregulated genes were subsequently visualized using a heatmap generated with the pheatmap package.

### 2.4 Weighted gene co-expression network analysis

A WGCNA was performed using the set of differentially expressed genes (DEGs) derived from the integrated, batch-corrected dataset (*n* = 103 samples). First, hierarchical clustering was conducted using average linkage to detect outlier samples. Four samples—GSM416554, GSM416550, GSM416535, and GSM416539—were excluded from subsequent analyses based on their extreme dissimilarity to the main cluster. This filtering step ensured a more coherent and reliable input dataset for network construction.

Next, the pickSoftThreshold function in the WGCNA package was used to identify an optimal soft thresholding power for the network. The power value of 6 was selected, as it satisfied the approximate scale-free topology criterion and yielded a high mean connectivity that balanced sensitivity and specificity in module detection.

Using a power of 6, the network was built by calculating pairwise correlations between the expression profiles of the DEGs and transforming them into a weighted adjacency matrix. The minimum module size (minModuleSize) was set to 40 to exclude small, potentially spurious modules, and modules with similar eigengenes were merged using a mergeCutHeight of 0.25. Each module was assigned a unique color identifier for clarity. Ultimately, this clustering procedure yielded two main co-expression modules.

Module–trait relationships were evaluated by correlating each module eigengene with the sample classification (healthy vs. ischemic stroke). Modules that did not correlate significantly with the trait were grouped into the “gray” category and excluded from further consideration. Genes within the modules of interest were assessed for both module membership (MM) and their involvement in the ERK pathway. The ggvenn package was employed to intersect module-member genes with ERK-related genes, identifying the most relevant candidates—termed “key genes”—for subsequent analyses.

### 2.5 Gene ontology enrichment analysis

Gene Ontology (GO) enrichment analysis was conducted to characterize the biological roles of the gene signature ERK (GSERK) genes. The clusterProfiler package and org. Hs.eg.db in R were used to calculate enrichment scores across three GO domains: biological process (BP), cellular component (CC), and molecular function (MF). For the BP and MF categories, genes were considered significantly enriched if they met thresholds of *p*-value < 0.05 and *q-*value cut off < 0.05. Because GSERK genes showed relatively weaker enrichment in the CC domain, we relaxed the criteria to *p*-value < 0.1 and *q*-value cut off < 0.1 for CC enrichment analyses. Enrichment results were visualized using bubble plots and circle plots to highlight the most prominently enriched GO terms.

### 2.6 Protein–protein interaction network construction

The identified GSERK genes were uploaded to the STRING database^3^ to predict protein–protein interactions (PPI). The resulting network was exported and further visualized in Cytoscape, where edges represent putative functional or physical associations among the GSERK proteins.

### 2.7 Hub gene screening by multiple algorithms

To prioritize key genes within the GSERK network, the cytoHubba plug-in in Cytoscape was employed. The computational algorithms were applied sequentially to rank the genes by their topological properties. Any gene not ranked as a hub by any of these methods was excluded from final consideration.

### 2.8 Differential expression analysis of hub GSERK genes

The hub GSERK genes were selected for in-depth expression profiling in ischemic stroke relative to healthy control samples. Expression data for these genes were extracted from both the complete dataset (“total sample set”) and an independent validation cohort. Boxplots were generated with ggpubr and forcats in R language, stratifying samples into ischemic stroke versus healthy control groups. Statistical significance between the two groups was determined using appropriate *post hoc* tests (e.g., *t*-tests or non-parametric equivalents), with *p*-values adjusted for multiple comparisons where necessary.

### 2.9 Receiver operating characteristic analysis

To evaluate the potential diagnostic utility of each hub GSERK gene, ROC curves were plotted and area under the curve (AUC) values were calculated using the pROC package. ROC analysis was performed separately for the total sample set and the validation cohort, with higher AUC values indicating stronger discriminatory power between IS and healthy subjects.

### 2.10 Machine learning–based feature selection

To robustly discern key biomarkers for ischemic stroke, four complementary machine learning algorithms were applied to the gene expression dataset. First, the Boruta algorithm was used to iteratively assess feature relevance by comparing actual predictor importance scores to those of randomized features. Second, a support vector machine (SVM) approach was used with a recursive feature elimination strategy, wherein features were iteratively removed based on their contribution to classification accuracy. Third, a least absolute shrinkage and selection operator (LASSO) model was implemented to impose an L1 penalty on regression coefficients, effectively shrinking the influence of less informative genes and retaining only the most predictive variables. Finally, a random forest classifier evaluated feature importance via the mean decrease in the Gini index, prioritizing features with the highest impact on classification purity at each node.

To maximize predictive power and minimize algorithm-specific biases, we compared the feature sets identified by each of the four methods. The final gene list was derived by taking the intersection of all selected features, thereby pinpointing genes that consistently emerged as top predictors across multiple machine learning paradigms.

### 2.11 Sample stratification and immune landscape profiling

To delineate immune heterogeneity in ischemic stroke, we stratified the harmonized dataset (*n* = 103) into two consensus clusters (*k* = 2) using *K*-means clustering with 1,000 iterations and Euclidean distance. Cluster stability was validated via consensus matrix analysis and cumulative distribution function (CDF) curves. Immune cell infiltration was quantified via single-sample gene set enrichment analysis (ssGSEA) using 28 immune cell type-specific gene sets. Stromal and immune scores were computed using the ESTIMATE algorithm, and inter-cluster differences were assessed via Wilcoxon rank-sum tests.

### 2.12 Predictive modeling and risk stratification

To establish a clinically translatable risk assessment framework for ischemic stroke, we constructed a nomogram integrating the GSERK hub genes using multivariable logistic regression. Gene expression values were standardized (*z*-scores) and assigned weighted points proportional to their regression coefficients. The model was validated via bootstrapping (1,000 resamples) to estimate calibration and discrimination metrics. Odds ratios (ORs) and 95% confidence intervals (CIs) for each gene were derived from univariate and multivariate analyses. Statistical significance was assessed using Wald tests, with *p* < 0.05 considered significant.

### 2.13 Western blot analysis

To evaluate the expression levels of ERK pathway–related proteins DUSP1 and GADD45A under hypoxia/reoxygenation (H/R) conditions, SH-SY5Y neuroblastoma cells were cultured and subjected to the following experimental treatments: (1) control group: Cells were maintained under normoxic conditions (21% O₂, 5% CO₂) for the full duration; (2) H/R group: cells were exposed to hypoxia (1% O₂, 5% CO₂, 94% N₂) for 6 h, followed by reoxygenation under normoxic conditions (21% O₂) for 24 h.

Following treatment, total protein was extracted using RIPA buffer supplemented with protease and phosphatase inhibitors. Protein concentrations were quantified using the BCA protein assay kit (Thermo Fisher Scientific, United States). Equal amounts of protein (20–30 μg) were separated on SDS-PAGE gels and transferred onto PVDF membranes. Membranes were blocked in 5% non-fat milk and incubated overnight at 4°C with the following primary antibodies: anti-DUSP1 (1:1000, Thermo Fisher Scientific, TA890036), anti-GADD45A (1:1000, Abcam, ab180768), and anti-GAPDH (1:5000, Thermo Fisher Scientific, MA5-15738) as a loading control. After washing, membranes were incubated with HRP-conjugated secondary antibodies (1:5000) for 1 h at room temperature. Protein bands were visualized using ECL detection reagents and quantified by ImageJ software. Relative expression levels of DUSP1 and GADD45A were normalized to GAPDH.

## 3 Results

### 3.1 Batch correction harmonizes ischemic stroke transcriptomes

**Figure 1:**
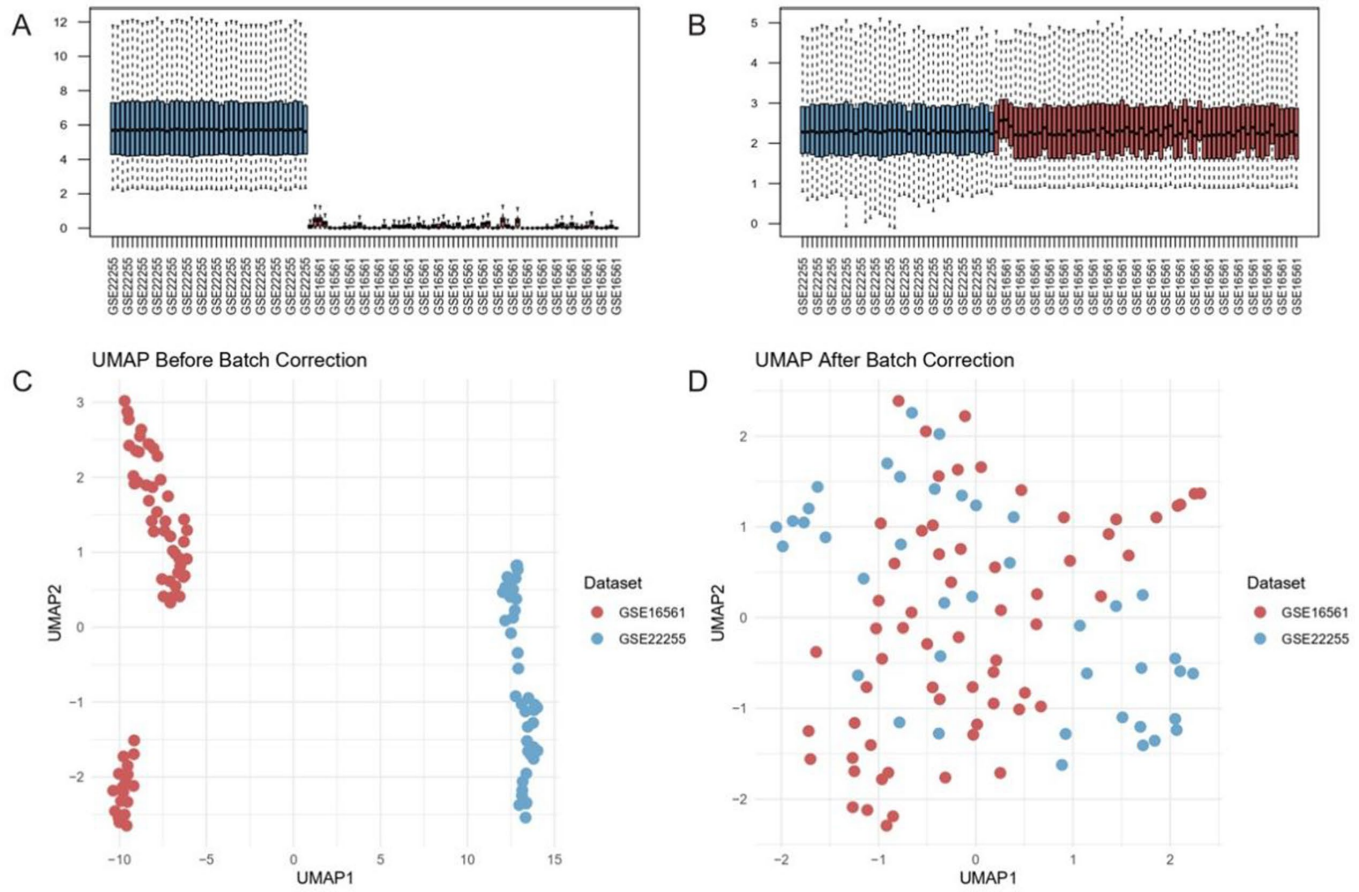
Batch effect correction harmonizes transcriptomic datasets GSE16561 and GSE22255. **(A,B)** Distribution of median expression values before **(A)** and after **(B)** batch correction. Post-correction medians align near the dashed diagonal, indicating reduced inter-dataset variability. **(C,D)** UMAP projections of samples prior to **(C)** and following **(D)** correction. The pronounced separation by dataset in the uncorrected data **(C)** is substantially diminished post-correction **(D)**, reflecting effective removal of batch-driven clustering.

To evaluate the effectiveness of batch effect correction across two ischemic stroke transcriptomic datasets (GSE16561 and GSE22255), we assessed their expression distribution patterns before and after data harmonization. Principal component analysis (PCA) revealed strong dataset-specific clustering in the uncorrected data, along with divergent median expression values (Figure 1A). After applying a robust batch correction algorithm, these distributions converged substantially, and median values aligned more closely, indicating effective mitigation of inter-dataset variability (Figure 1B).

We further validated these findings using UMAP. In the uncorrected data, GSE16561 and GSE22255 occupied distinct, non-overlapping clusters, reflecting pronounced technical differences between the cohorts (Figure 1C). Post-correction, samples from both datasets became interspersed, suggesting that batch-driven artifacts were minimized while preserving underlying biological variation (Figure 1D). These observations underscore the importance of implementing rigorous batch correction strategies for integrative analyses of heterogeneous genomic datasets in ischemic stroke research.

### 3.2 Differential expression analysis between stroke cases and controls within the combined GSE16561 and GSE22255

**Figure 2:**
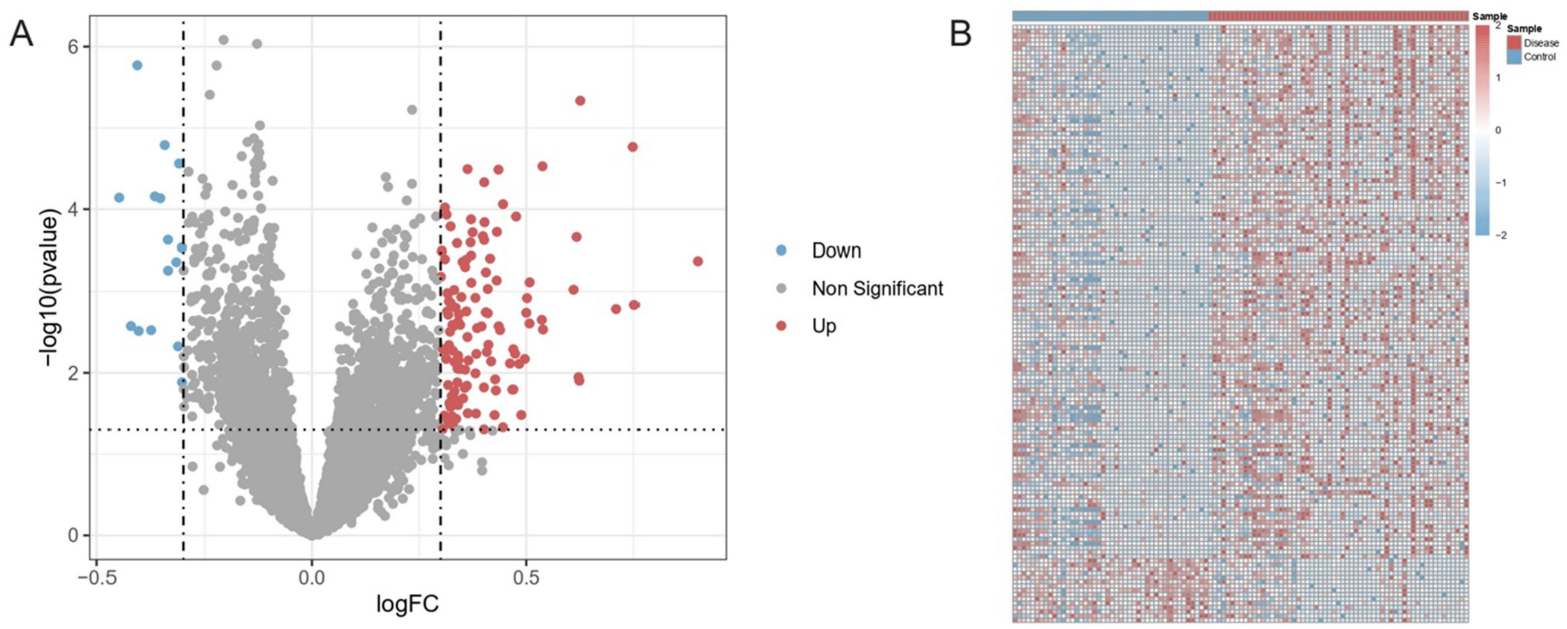
Differential gene expression between ischemic stroke and healthy samples. **(A)** Volcano plot of DEGs (adjusted *p* < 0.05, |log2FoldChange| > 0.3). Vertical dashed lines: fold change thresholds; horizontal line: significance cutoff. Red/blue points: upregulated/downregulated genes. **(B)** Hierarchically clustered heatmap of DEGs across samples. Rows: genes; columns: samples (blue: controls; red: ischemic stroke). Color scale reflects normalized expression (low: blue; high: red).

From the total of 103 samples subjected to differential expression analysis (44 healthy and 59 diseased), 140 genes met the significance criteria (adjusted *p*-value <0.05 and |log2FC| > 0.3). Of these, 125 genes were upregulated in ischemic stroke samples compared with healthy controls, whereas 15 genes were downregulated. The volcano plot revealed that distribution of DEGs in Figure 2A. Additionally, heatmap analysis of the DEGs further illustrated distinct expression profiles between healthy controls and ischemic stroke samples (Figure 2B).

### 3.3 Weighted co-expression network analysis unveils ERK pathway-associated modules in ischemic stroke

**Figure 3:**
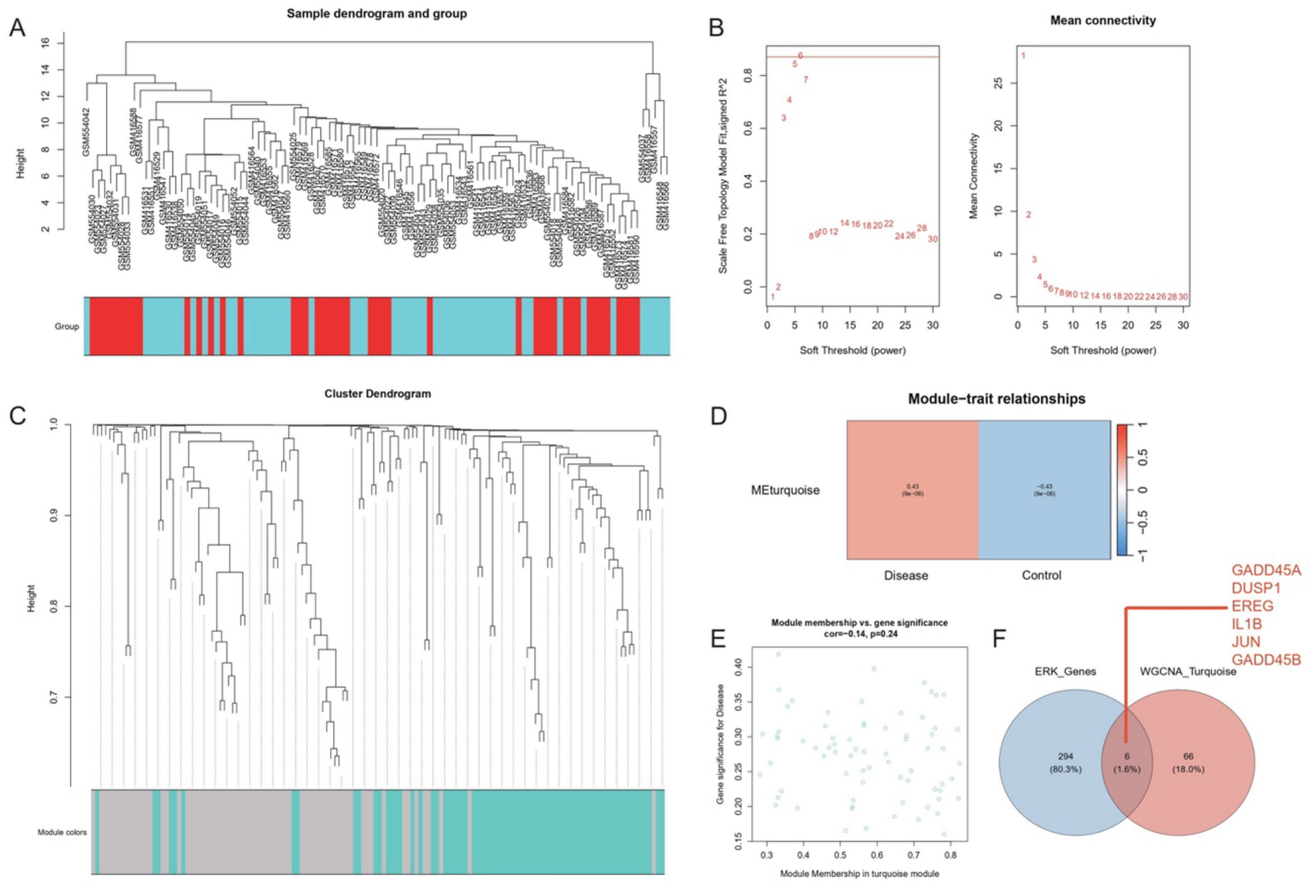
Weighted gene co-expression network analysis (WGCNA) identifies disease-relevant co-expression modules and ERK pathway hubs. **(A)** Hierarchical clustering of samples revealed four outlier samples, which were excluded to minimize noise. **(B)** Scale-free topology analysis determined an optimal soft threshold power of 6 (left: signed *R*^2^; right: mean connectivity). **(C)** Dynamic tree cutting (minModuleSize = 40) and module merging (mergeCutHeight = 0.25) yielded two major co-expression modules (color-coded). **(D)** Heatmap of module–trait correlations (rows: modules; columns: phenotype) highlighted the turquoise module’s strong association with ischemic stroke (*p* < 0.001). **(E)** Scatter plot of module membership (turquoise module) versus gene significance for disease status revealed a negative correlation (Pearson *r* = −0.14, *p* = 0.24), suggesting regulatory roles in pathogenesis. **(F)** Venn diagram identified six ERK pathway genes (*GADD45A*, *DUSP1*, *EREG*, *IL1B*, *JUN*, *GADD45B*) intersecting with the turquoise module.

Hierarchical clustering revealed four outlier samples (GSM416554, GSM416550, GSM416535, GSM416539), which were removed to ensure the resulting network captured genuine co-expression relationships rather than artifacts from disparate data points (Figure 3A). By systematically evaluating the scale-free topology fit at different power values, a soft threshold of 6 was chosen (Figure 3B). Under this optimal threshold, co-expression modules were identified using dynamic tree cutting with minModuleSize set to 40. Subsequent merging of closely related modules (mergeCutHeight = 0.25) resulted in two major co-expression modules, each assigned a distinct color tag (Figure 3C). Correlation analysis between module eigengenes and sample phenotypes indicated that at least one of the resulting modules exhibited strong relevance to the ischemic stroke phenotype. A heatmap of module–trait correlations confirmed this module as a primary focus of further investigation (Figure 3D).

A scatter plot of module membership versus gene significance (or correlation to the trait) indicated a predominantly negative correlation for genes in the key module (Figure 3E), suggesting potential modulatory or inhibitory roles in the disease process. Using the ggvenn package, six ERK-related genes (*GADD45A*, *DUSP1*, *EREG*, IL1beta, *JUN*, and *GADD45B*) were found at the intersection of module membership and known ERK pathway components (Figure 3F). These genes, collectively referred to as “gene signature ERK pathway (GSERK),” emerged as critical hubs for further functional validation, laying the groundwork for subsequent exploration of their roles in ischemic stroke pathophysiology.

### 3.4 GO enrichment analysis of GSERK genes

Under the specified thresholds (*p* < 0.05, *q* < 0.05 for BP and MF), the GSERK gene set displayed significant enrichment in 163 biological processes and 13 molecular functions. For the cellular component category, nine enriched terms were identified using a more permissive cutoff (*p* < 0.1, *q* < 0.1).

**Figure 4:**
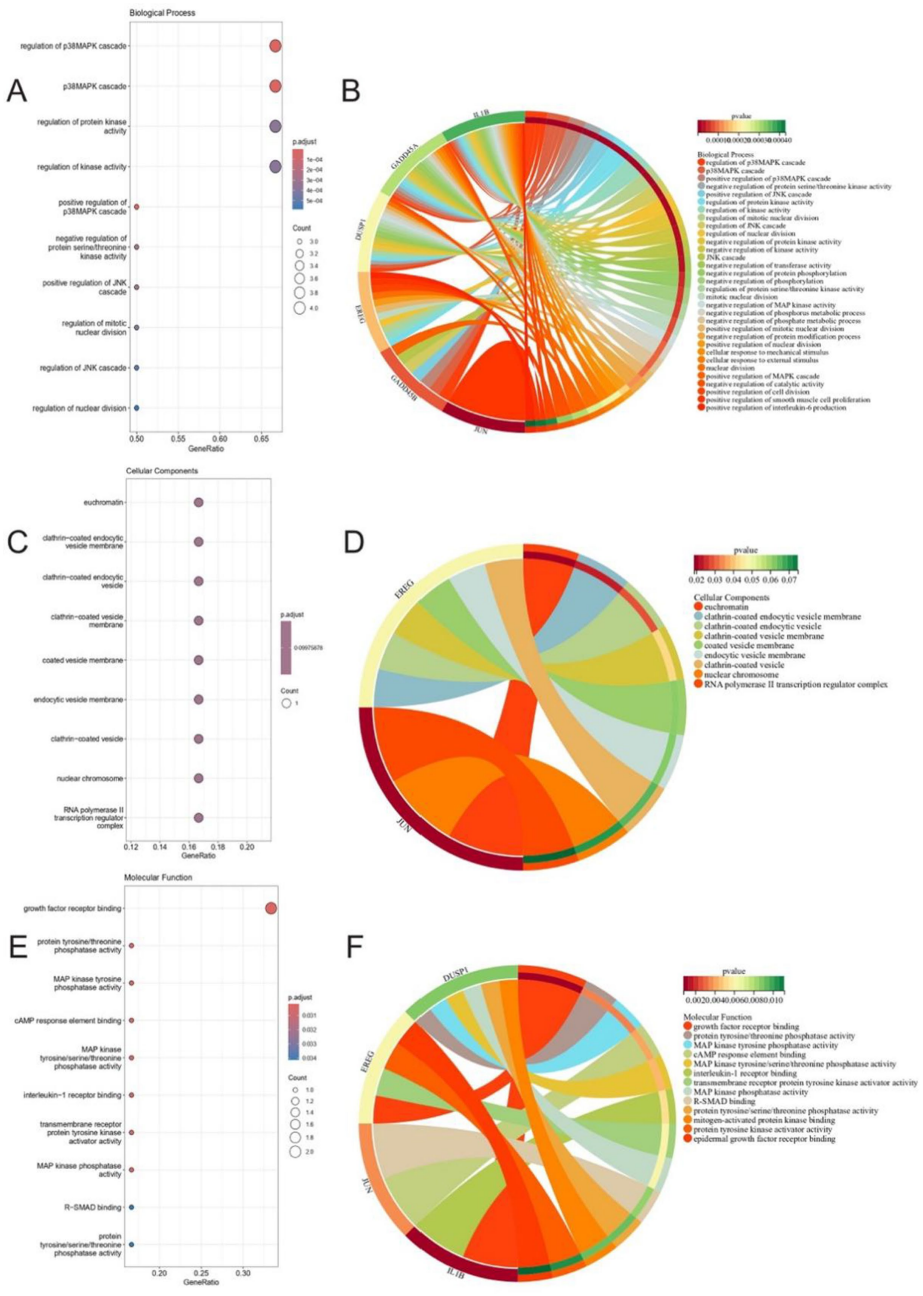
Gene ontology enrichment analysis highlights GSERK genes’ roles in MAPK signaling and growth factor regulation. **(A,B)** Enriched biological processes (BP) under stringent thresholds (*p* < 0.01, *q* < 0.01). Top terms include regulation of p38MAPK cascade and regulation of kinase activity, with 5/6 GSERK genes (*GADD45A*, *DUSP1*, *IL1B*, *GADD45B*, *EREG*) implicated. Bars represent gene ratios; color intensity reflects −log10 (p.adjust). **(C,D)** Cellular component (CC) enrichment under relaxed thresholds (*p* < 0.1, *q* < 0.1). Terms such as clathrin-coated vesicle membrane and euchromatin suggest subcellular localization dynamics. **(E,F)** Molecular function (MF) terms (thresholds: *p* < 0.05, *q* < 0.05). Growth factor receptor binding (EREG and IL1B) and MAP kinase phosphatase activity (DUSP1) were most enriched. Dot size indicates gene count; color scale corresponds to statistical significance.

In biological process enrichment, a total of 32 BP terms passed a more stringent cutoff of *p* < 0.01 and *q* < 0.01 (Figures 4A,B). Notably, GSERK demonstrated the strongest enrichment in regulation of p38MAPK cascade, p38MAPK cascade itself, regulation of protein kinase activity, and regulation of kinase activity. Each of these processes involved five of the six GSERK members (*GADD45A*, *DUSP1*, *IL1B*, *GADD45B*, and *EREG*). These findings underscore a mechanistic link between GSERK and MAPK-driven signaling pathways, which are known to be pivotal in ischemic stroke pathophysiology.

While GSERK genes did not exhibit strong enrichment in the CC category under the standard significance thresholds, we identified nine enriched cellular components at *p* < 0.1 and *q* < 0.1 (Figures 4C,D). This less stringent criterion allowed the capture of potential CC associations that may still contribute to the spatial dynamics of these critical genes within the cell.

Among the 13 significantly enriched MF terms (Figures 4E,F), growth factor receptor binding pathway showed the highest enrichment ratio under MF enrichment. *EREG* and *IL1B* were specifically implicated in this functional category, suggesting they may modulate key receptor-mediated signaling events pertinent to ERK pathway activation in ischemic stroke. Collectively, these GO analyses point to a central role for GSERK genes in modulating kinase-related signaling cascades and growth factor receptor interactions, further highlighting their potential as therapeutic targets or biomarkers in ischemic stroke research.

### 3.5 GSERK protein–protein interaction network and identification of hub GSERK genes

**Figure 5:**
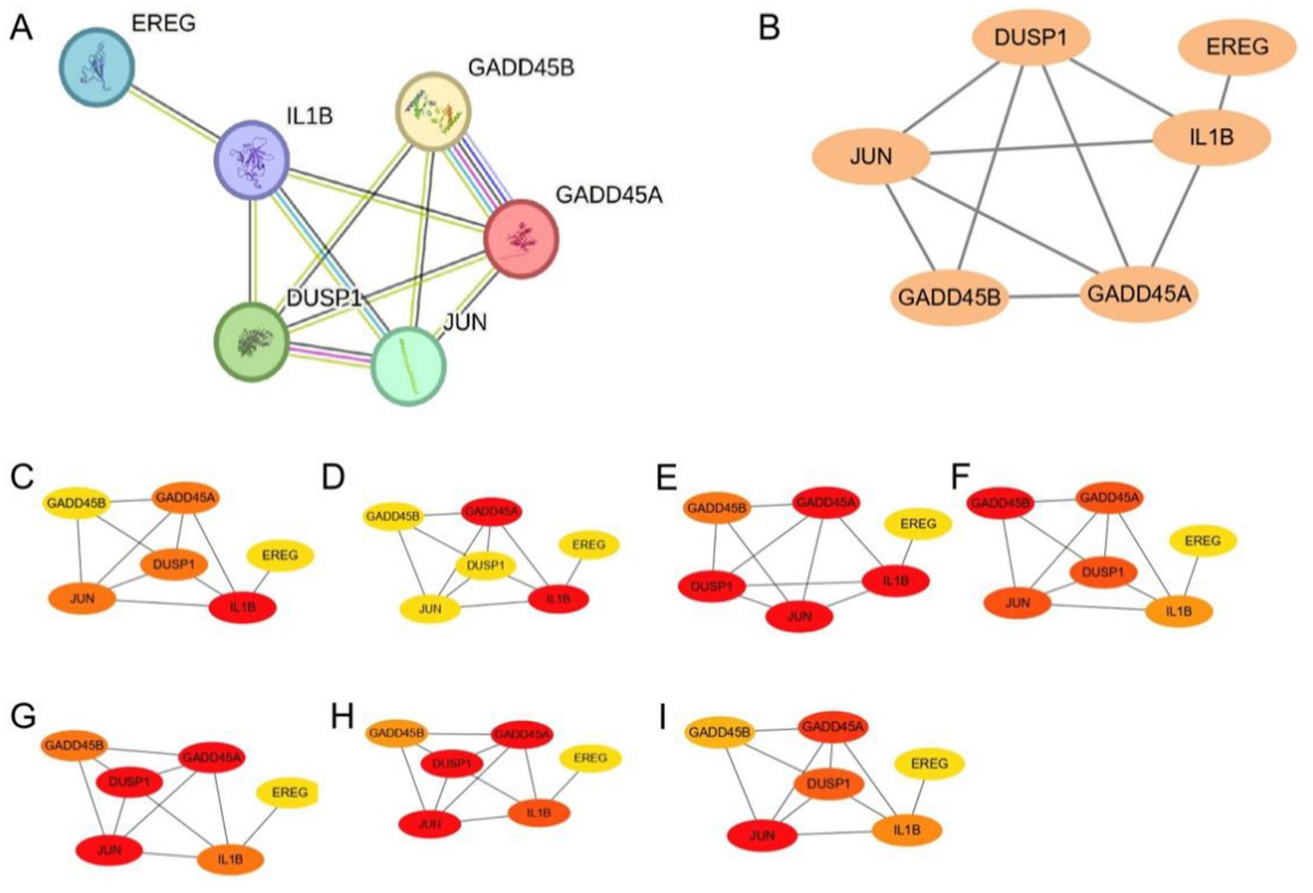
GSERK protein–protein interaction network and hub gene analysis. **(A)** STRING-derived PPI network of six GSERK proteins (GADD45A, DUSP1, EREG, IL1B, JUN, and GADD45B). Node size and edge thickness indicate predicted interaction strength. **(B)** Cytoscape visualization of the same network with manual layout adjustments for clarity. **(C–I)** Color-coded hub gene rankings generated by each of the seven algorithms in the cytoHubba plug-in. Red nodes represent the highest-ranking genes, whereas yellow nodes indicate lower ranks. EREG was not identified as a hub by any algorithm and was thus excluded from the final hub GSERK set.

The STRING-based PPI network (Figure 5A) highlighted direct and indirect links among the six GSERK proteins, indicating extensive interconnectivity within stress- and inflammation-related pathways. Cytoscape-based refinement (Figure 5B) enabled more detailed visualization, revealing strong central positioning for five of the GSERK genes (*GADD45A*, *DUSP1*, *IL1B*, *JUN*, *GADD45B*), whereas EREG showed fewer high-confidence interactions.

Applying seven distinct cytoHubba algorithms, EREG did not meet the criteria for a hub gene in any of the methods employed. Consequently, the remaining five genes were designated as the hub GSERK members: *GADD45A*, *DUSP1*, *IL1B*, *JUN*, and *GADD45B*. Color-coded network diagrams for each algorithm (Figures 5C–I) consistently highlighted these five genes as central nodes, underscoring their potential significance in modulating ERK-related signaling cascades implicated in ischemic stroke pathogenesis.

### 3.6 Differential expression and diagnostic performance of hub GSERK genes

**Figure 6:**
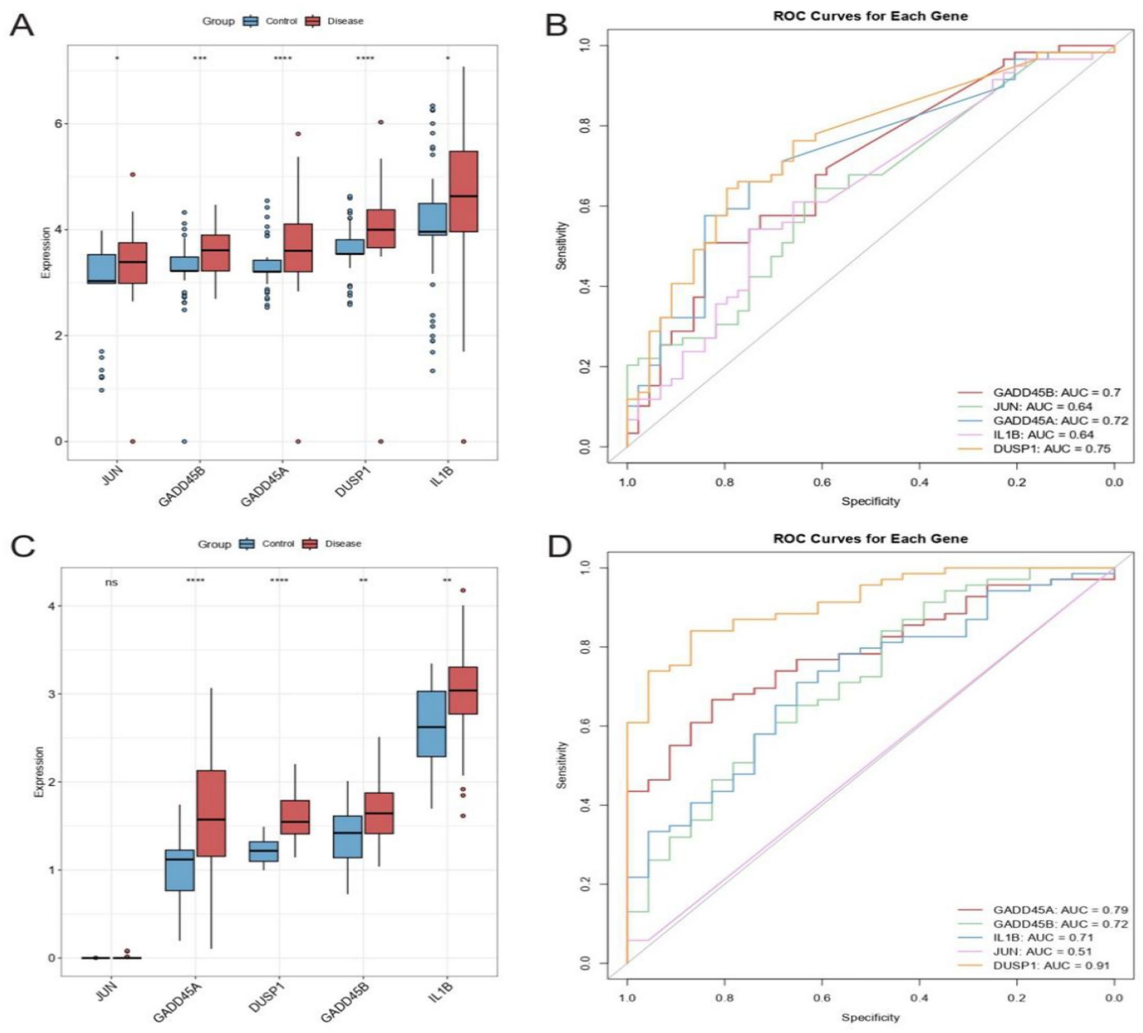
Differential expression and diagnostic utility of hub GSERK genes. **(A)** Boxplots comparing the expression of *JUN*, *GADD45B*, *GADD45A*, *DUSP1*, and *IL1B* in the total sample set (ischemic stroke vs. healthy). **(B)** ROC curves and corresponding AUC values for the total sample set, demonstrating moderate diagnostic capacity for DUSP1, GADD45A, and GADD45B. **(C)** Boxplots of gene expression in the validation cohort, showing significant upregulation of *GADD45A*, *DUSP1*, *GADD45B*, and IL1B in ischemic stroke samples, whereas JUN levels did not differ significantly. **(D)** ROC curves for the validation cohort underscore the strong predictive performance of DUSP1, followed by GADD45A and GADD45B, consistent with findings from the total sample set.

In the total sample set, all five hub GSERK genes were upregulated in IS samples compared with healthy controls (Figure 6A). Of particular note, *GADD45A*, *DUSP1*, and *GADD45B* showed marked differences (*p* < 0.001) between the two groups. In the validation cohort (Figure 6C), four genes (*GADD45A*, *DUSP1*, *GADD45B*, and *IL1B*) were significantly upregulated in IS relative to healthy controls (*p* < 0.001), whereas *JUN* did not display a statistically significant difference. These findings highlight potential roles for *GADD45A*, *DUSP1*, *GADD45B*, and *IL1B* as robust markers of ischemic stroke.

The ROC curve analysis in the total sample set revealed moderate discriminatory capacity for *DUSP1* (AUC = 0.75), *GADD45A* (AUC = 0.72), and *GADD45B* (AUC = 0.70), suggesting these genes may serve as potential diagnostic biomarkers for ischemic stroke (Figure 6B). Consistent with these results, the validation cohort showed high diagnostic performance for *DUSP1* (AUC = 0.91), followed by *GADD45A* (AUC = 0.79) and *GADD45B* (AUC = 0.72) (Figure 6D). Collectively, these results support the potential of DUSP1, GADD45A, and GADD45B as useful biomarkers for ischemic stroke, warranting further clinical validation.

### 3.7 Identification of common genes by four machine learning approaches

**Figure 7:**
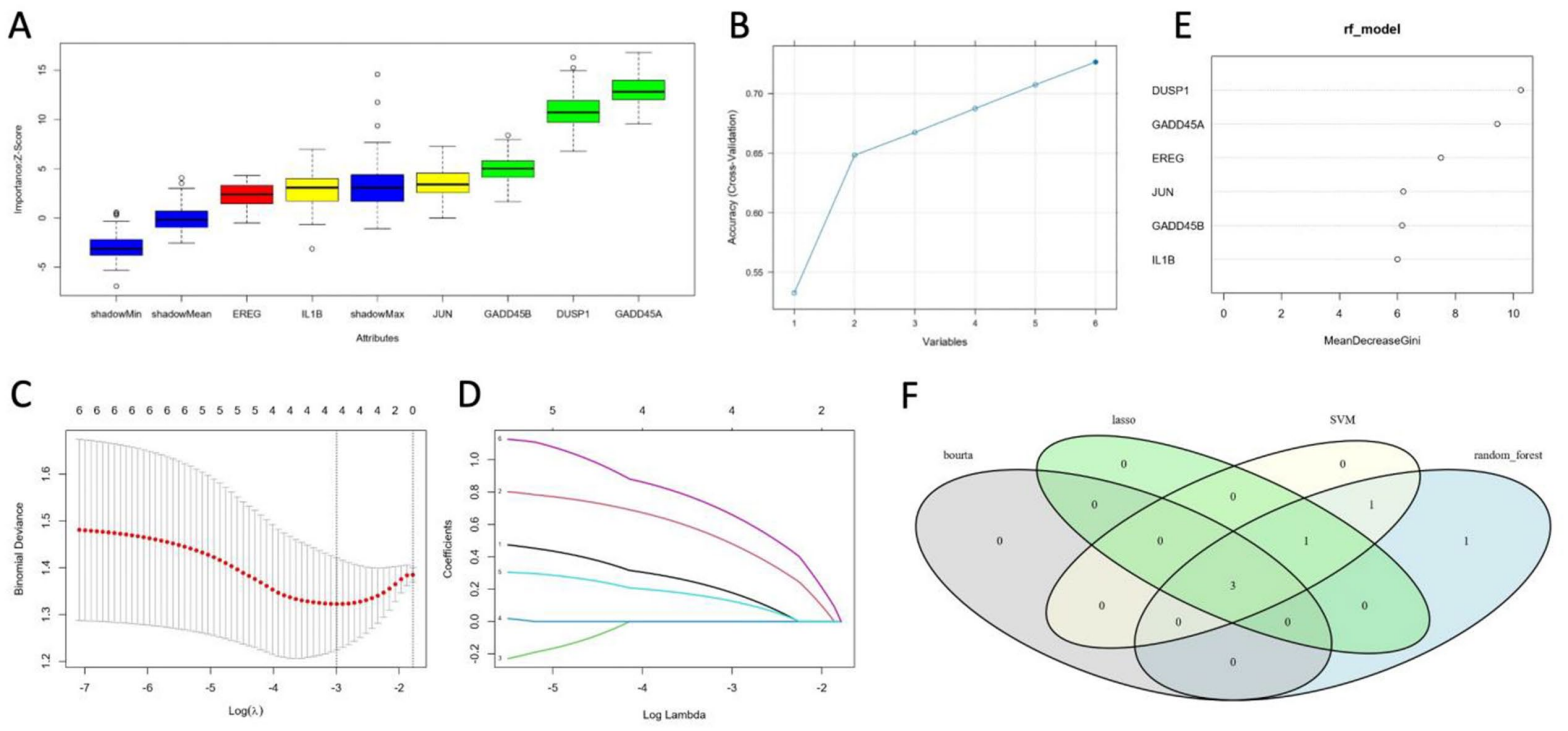
Identification of key candidate genes using four machine learning methods. **(A)** Boruta algorithm results, showing the distribution of importance scores (Z-scores) for each candidate feature, as well as “shadow” features used for reference. **(B)** SVM-based feature selection curve illustrating the incremental accuracy gains as different subsets of features are retained or eliminated. **(C,D)** LASSO path plots demonstrating how coefficients of candidate features shrink toward zero at increasing penalty values (log *λ*). **(E)** Random forest feature importance scores, measured by the mean decrease in Gini index. **(F)** Venn diagram depicting the intersection of genes identified by the four algorithms. *GADD45A*, *DUSP1*, and *GADD45B* appear in all feature sets, indicating a high level of consensus regarding their predictive value.

All four machine learning algorithms identified a partially overlapping set of candidate biomarkers, reflecting each method’s distinct way of handling noise and collinearity in the data (Figures 7A–E). Upon intersecting the results (Figure 7F), three genes—*GADD45A*, *DUSP1*, and *GADD45B*—were consistently ranked as important across Boruta, SVM, LASSO, and random forest analyses. These robust markers thus hold particular promise for further validation and potential translational applications in ischemic stroke research.

### 3.8 Immune heterogeneity and stromal remodeling define ischemic stroke subgroups

**Figure 8:**
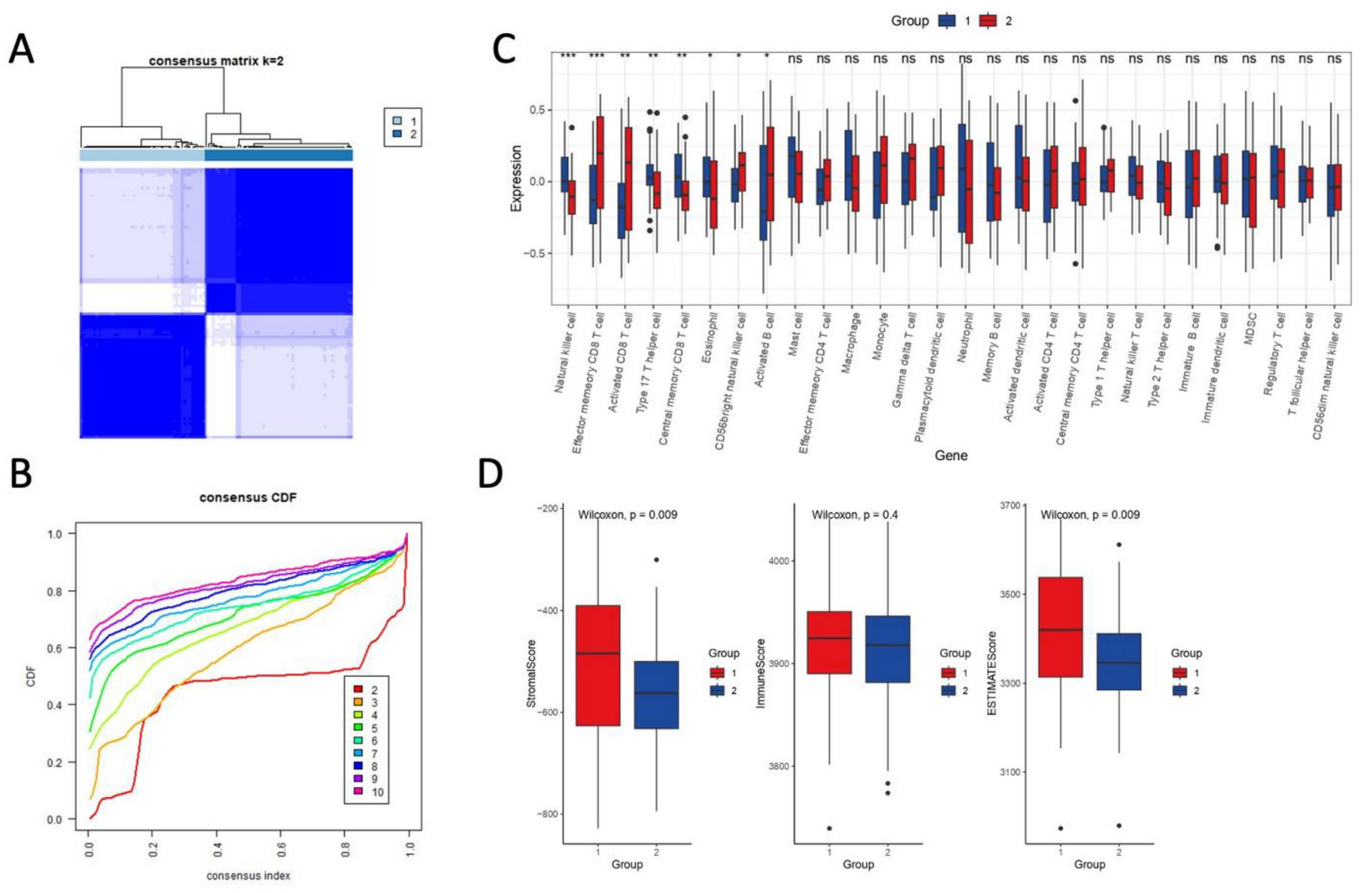
Consensus clustering and immune-stromal profiling define ischemic stroke subgroups. **(A)** Consensus matrix heatmap (*k* = 2) demonstrating robust sample clustering. Rows and columns represent samples, with color intensity reflecting pairwise consensus values (0–1; white: low consensus, dark blue: high consensus). Clusters were generated using *K*-means (Euclidean distance, 1,000 iterations). **(B)** Cumulative distribution function (CDF) curves for consensus clustering. The area under the curve indicates high cluster stability. Dashed lines denote consensus index distributions for *k* = 2–10. **(C)** Single-sample gene set enrichment analysis (ssGSEA) heatmap of 28 immune cell types across subgroups (Subgroup 1: left; Subgroup 2: right). Color scale reflects normalized enrichment scores (blue: low, red: high). Asterisks mark significant differences (*p* < 0.05, Wilcoxon rank-sum test). **(D)** Boxplots comparing ImmuneScore, StromalScore, and ESTIMATEScore (ESTIMATE algorithm) between subgroups.

To resolve immune-stromal heterogeneity in ischemic stroke, we stratified the harmonized cohort (*n* = 103) into two molecular subgroups using *K*-means clustering (*k* = 2, Figure 8A). Consensus matrix analysis confirmed robust cluster stability, as indicated by an area under the CDF curve (Figure 8B). Subgroup 2 exhibited a pronounced hyperinflammatory phenotype, characterized by elevated infiltration of effector memory CD8^+^ T cells, activated CD8^+^ T cells, macrophages, and neutrophils. In contrast, Subgroup 1 showed enrichment for natural killer cells, T helper 17 (Th17) cells, and central memory CD8^+^ T cells (Figure 8C). These findings suggest divergent immune microenvironments that could influence therapeutic responsiveness.

Application of the ESTIMATE algorithm further distinguished the two subgroups. Subgroup 1 demonstrated significantly higher StromalScores (*p* = 0.009) and ESTIMATEScores (*p* = 0.009), indicative of active immune-stromal crosstalk (Figure 8D). In contrast, Subgroup 1 also showed muted stromal remodeling (*p* = 0.4), potentially reflecting adaptive mechanisms to aiming at mitigating ischemic damage. Notably, these subgroup-specific profiles align with ERK pathway dynamics, wherein hyperinflammation may exacerbate oxidative stress, whereas more quiescent stromal remodeling could temper reparative signaling.

### 3.9 A GSERK-based nomogram predicts ischemic stroke risk and highlights age as a significant factor

**Figure 9:**
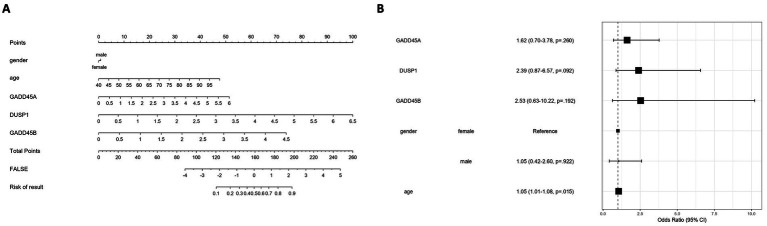
Development and evaluation of a GSERK-based nomogram for ischemic stroke risk prediction. **(A)** A nomogram was constructed based on multivariable logistic regression incorporating three ERK pathway-associated genes (*GADD45A*, *DUSP1*, and *GADD45B*), along with clinical variables (age and gender). Each predictor is assigned a weighted point value, with total points corresponding to an estimated probability of ischemic stroke. **(B)** Forest plot summarizing the odds ratios (OR) and 95% confidence intervals (CI) from the multivariate logistic regression model. Among the variables, age was a statistically significant independent predictor of stroke risk (OR = 1.05, 95% CI: 1.01–1.08, *p* = 0.015). *DUSP1* and *GADD45B* demonstrated trends toward association (OR = 2.39 and 2.53, respectively), though not statistically significant. Gender and GADD45A were not significantly associated with outcome.

To translate GSERK gene signatures into a clinically applicable model, we constructed a nomogram incorporating the expression levels of GADD45A, DUSP1, and GADD45B, along with age and gender as clinical covariates (Figure 9A). Each variable contributed a weighted score based on multivariable logistic regression coefficients, and the total score was mapped to an estimated stroke risk probability (ranging from 0.1 to 0.9). The nomogram offers a visual, interpretable tool for individualized risk assessment. Multivariate logistic regression analysis (Figure 9B) revealed that among the molecular variables, *DUSP1* (OR = 2.39, 95% CI: 0.87–6.57; *p* = 0.092) and *GADD45B* (OR = 2.53, 95% CI: 0.63–10.22; *p* = 0.192) showed a trend toward stroke association, though neither reached statistical significance. *GADD45A* exhibited a weaker effect (OR = 1.92, 95% CI: 0.70–5.71; *p* = 0.298). Notably, age emerged as a statistically significant predictor of stroke risk (OR = 1.05, 95% CI: 1.01–1.08; *p* = 0.015), highlighting the importance of integrating clinical parameters into molecular models. Gender was not significantly associated with outcome (male vs. female: OR = 1.05, *p* = 0.922). These results suggest that while ERK-associated genes, particularly *DUSP1* and *GADD45B*, may contribute to ischemic stroke susceptibility, age remains the dominant independent predictor in this integrative model. Further validation in larger cohorts may assist clarify the prognostic relevance of GSERK components.

### 3.10 DUSP1 and GADD45A are upregulated in response to hypoxia/reoxygenation in SH-SY5Y cells

**Figure 10:**
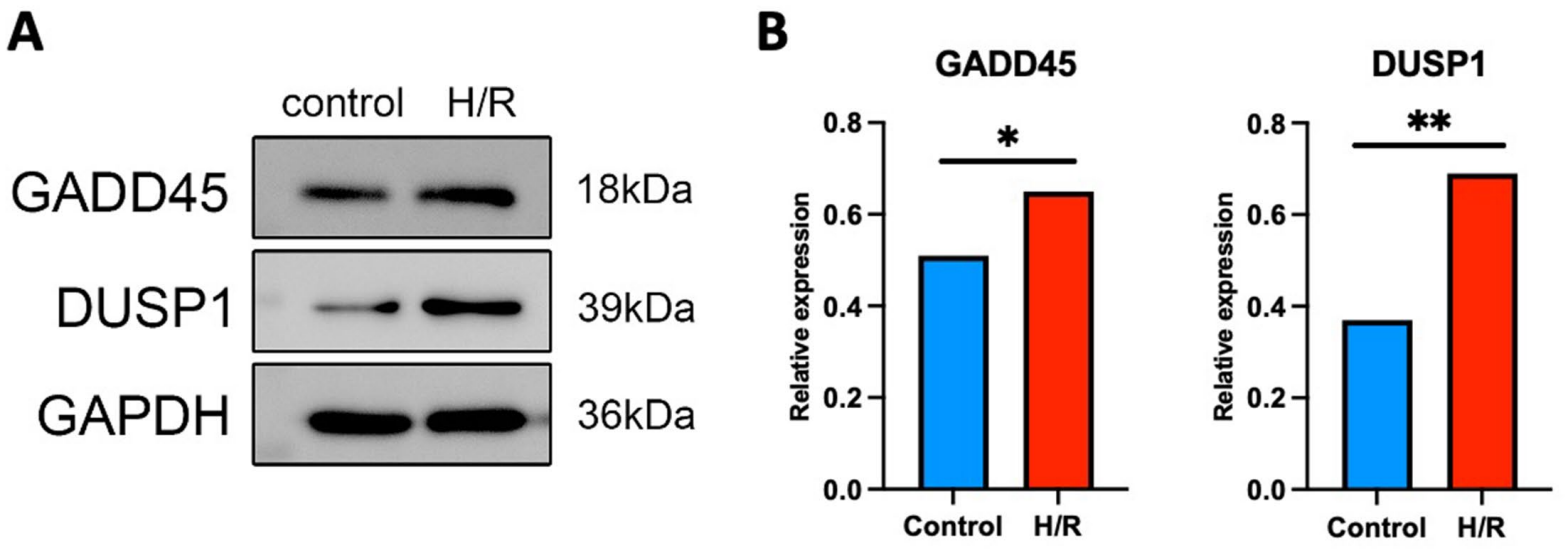
Hypoxia/reoxygenation (H/R) induces upregulation of DUSP1 and GADD45A protein expression in SH-SY5Y cells. **(A)** Representative Western blot images showing the expression levels of GADD45A and DUSP1 under normoxic control and H/R conditions. GAPDH was used as a loading control. **(B)** Quantification of relative protein expression levels normalized to GAPDH. H/R treatment significantly increased DUSP1 and GADD45A expression compared to controls. Data are presented as mean ± SEM from three independent experiments. ^*^p < 0.05, ^**^p < 0.01, Student’s *t*-test.

Western blot analysis revealed that both DUSP1 and GADD45A protein levels were significantly upregulated in SH-SY5Y cells subjected to H/R compared to normoxic controls (Figure 10A). Densitometric quantification demonstrated an increase in DUSP1 expression (*p* < 0.01, Figure 10B) and GADD45A expression (*p* < 0.05, Figure 10B), normalized to GAPDH. These findings support the transcriptomic prediction that ERK-associated genes are responsive to ischemic-like stress and may play a regulatory role in the cellular response to oxidative injury.

## 4 Discussion

In this study, we integrated heterogeneous ischemic stroke transcriptomic datasets (GSE16561 and GSE22255) through rigorous batch correction, substantially improving data coherence and enabling robust identification of disease-associated molecular signatures. Leveraging multiple bioinformatic approaches, we pinpointed a critical gene signature within the ERK pathway—termed GSERK—that prominently included *GADD45A*, *DUSP1*, and *GADD45B* as central modulators implicated in ischemic stroke pathogenesis. Furthermore, by employing immune-stromal profiling and consensus clustering analyses, we defined distinct ischemic stroke molecular subgroups characterized by differential immune cell infiltration and stromal remodeling. These findings not only underscore the biological heterogeneity of ischemic stroke but also suggest that subgroup-specific immune and ERK pathway dynamics may guide the development of personalized therapeutic strategies and more precise diagnostic tools.

Rigorous batch correction emerged as an indispensable step for integrating heterogeneous ischemic stroke transcriptomic datasets, underscoring its critical role in minimizing technical variability and enhancing biological interpretability. Before harmonization, our PCA and UMAP visualizations clearly demonstrated dataset-specific clustering, indicative of substantial batch-driven artifacts. After implementing robust batch correction methods, these technical biases were notably mitigated, as evidenced by improved overlap and coherent expression patterns between datasets. Such methodological rigor not only enabled precise identification of DEGs but also greatly enhanced our capacity to uncover genuine biological variation, rather than technical confounders. Consequently, downstream analyses, including WGCNA, accurately captured biologically meaningful modules closely associated with ischemic stroke pathophysiology, including ERK pathway-associated signatures. These results underscore the necessity of systematic data harmonization approaches when combining multiple genomic datasets, aligning with current best practices in bioinformatics research for complex diseases such as ischemic stroke (Leek et al., 2010; Johnson et al., 2007; Tran et al., 2020).

Our differential expression analysis highlighted 140 significantly altered genes between ischemic stroke and control samples, with a notable majority (125 genes) upregulated in stroke-affected tissues. Predominantly, these DEGs were inflammatory mediators, underscoring the profound involvement of inflammation in ischemic stroke pathogenesis and progression (Anrather and Iadecola, 2016; Iadecola et al., 2020). Such inflammatory signatures are consistent with well-documented evidence highlighting neuroinflammation as a central driver of ischemic injury, neuronal death, and exacerbation of neurological deficits (Kelly et al., 2021; Chamorro et al., 2016). Furthermore, our pathway enrichment analysis distinctly emphasized genes associated with the ERK/MAPK signaling pathways, particularly highlighting strong enrichment in processes regulating the p38MAPK cascade and kinase activity modulation. The MAPK signaling cascades, including ERK and p38MAPK pathways, have been extensively implicated in stroke-related inflammation, oxidative stress, neuronal apoptosis, and disruption of the blood–brain barrier integrity (Sun and Nan, 2017; Kong et al., 2019; Fann et al., 2018). Therefore, our findings support the hypothesis that MAPK signaling modulation may serve as a strategic therapeutic target to alleviate ischemic brain damage and improve clinical outcomes following stroke (Sun and Nan, 2016).

Our WGCNA provided robust insight into biologically meaningful modules closely associated with ischemic stroke pathology, especially those linked to ERK pathway signaling. Using this approach, we identified a distinct gene signature—termed GSERK—including *GADD45A*, *DUSP1*, *EREG*, *IL1B*, *JUN*, and *GADD45B* as central regulatory nodes within ischemic stroke-related co-expression networks. These genes have previously been implicated in multiple pathological contexts relevant to ischemic stroke. For instance, DUSP1 acts as a critical regulator of MAPK activity, exerting protective effects by attenuating inflammation-induced neuronal injury and apoptosis (Sun et al., 2021). GADD45A and GADD45B have been linked to stress responses and modulation of apoptosis, inflammation, and cell cycle arrest following neuronal damage (Liebermann and Hoffman, 2008; Grinan-Ferre et al., 2024). GADD45 proteins, including GADD45A and GADD45B, function as stress sensors through physical interactions with proteins involved in cell cycle regulation and stress responses (Liebermann and Hoffman, 2008). GADD45A is involved in cellular responses to genotoxic stress, which includes cell cycle checkpoints, DNA repair, and apoptosis (Zhan, 2005). In response to genotoxic stresses, GADD45A interacts with CDK1, leading to the dissociation of the CDK1-cyclin B1 complex and subsequent inhibition of CDK1 kinase activity, resulting in G2/M cell cycle arrest and cell growth suppression (Palomer et al., 2024). GADD45A can protect against H/R-induced apoptosis in human embryonic cardiomyocytes through the p38 MAPK signaling pathway (Xie et al., 2023). Additionally, previous study suggested that knockdown of GADD45B accelerates neuronal cell death and mitochondrial dysfunction (Cho et al., 2019). In non-neuronal cells, GADD45B interacts with regulatory factors and signaling pathways to control the cell cycle, DNA repair, and cell survival/apoptosis (Liu et al., 2009). GADD45B promotes cell apoptosis through the p38/MAPK pathway (Wang et al., 2021). Similarly, *IL1B* and *JUN* play central roles in amplifying inflammatory cascades, exacerbating neuronal death and worsening functional outcomes (Bodnar et al., 2021). Thus, identifying these genes as hub nodes highlights their biological significance as regulators of ERK pathway-mediated injury responses in ischemic stroke, positioning them as attractive targets for therapeutic intervention and diagnostic biomarker development.

Our GO enrichment analyses provided valuable functional insights into the roles played by GSERK genes in ischemic stroke pathophysiology. Particularly noteworthy was the significant enrichment observed in biological processes, such as the regulation of the p38 MAPK cascade, protein kinase activity modulation, and overall kinase regulation. These processes are crucial determinants of cellular responses during ischemic stress, influencing inflammation, oxidative stress, apoptosis, and subsequent tissue injury (Canovas and Nebreda, 2021). DUSP1, one of the central GSERK genes identified, encodes a dual-specificity phosphatase critically involved in attenuating excessive MAPK activation, thereby potentially limiting ischemia-induced neuroinflammation and neuronal cell death (Qin et al., 2023). Similarly, IL1B emerged as pivotal due to its established role in amplifying inflammatory cascades through activation of downstream MAPK signaling, contributing directly to secondary brain injury and edema formation (Thapa et al., 2021). Despite limited enrichment in cellular component categories, our results suggest that GSERK proteins could exhibit dynamic subcellular localization in response to ischemic stress. Future studies should investigate whether GSERK subcellular trafficking or compartment-specific activation contributes to differential cellular outcomes in ischemic stroke, thereby identifying additional therapeutic targets and deepening the mechanistic understanding of stroke pathogenesis.

Our network analyses further highlighted the clinical promise of GSERK hub genes as key therapeutic targets in ischemic stroke. PPI analyses delineated an interconnected network among the GSERK proteins, underscoring their collective involvement in stress-induced signaling and inflammatory cascades. Notably, EREG exhibited fewer high-confidence interactions and was thus deprioritized, allowing us to concentrate on the five core hub genes: *GADD45A*, *DUSP1*, *IL1B*, *JUN*, and *GADD45B*. These genes consistently emerged as central nodes across multiple cytoHubba algorithms, reinforcing their robustness as therapeutic targets (Szklarczyk et al., 2019; Doncheva et al., 2023). The central positions of DUSP1, GADD45A, and GADD45B within this network are particularly noteworthy, suggesting their influential roles in modulating MAPK-mediated neuronal injury, apoptotic pathways, and immune responses during ischemic events (Palomer et al., 2024; Cho et al., 2019). Given their reproducible significance across diverse bioinformatics and machine learning approaches, these genes warrant consideration for targeted pharmacological interventions aiming at mitigating ischemic brain injury. Additionally, we demonstrated substantial diagnostic and translational potential for GSERK biomarkers through rigorous differential expression and receiver operating characteristic (ROC) analyses. *DUSP1*, *GADD45A*, and *GADD45B* consistently showed robust discriminatory power, with validation cohorts yielding impressive AUC values of 0.91, 0.79, and 0.72, respectively. Such findings highlight the prospective utility of these biomarkers for early ischemic stroke detection, accurate patient stratification, and potentially as markers of therapeutic responsiveness (Dagonnier et al., 2021). Future studies should explore these genes in larger prospective cohorts and clinical trials to establish their clinical validity, ultimately translating genomic insights into improved patient outcomes.

Our classification of ischemic stroke samples into “hyperinflammatory” and “quiescent stromal” molecular subtypes revealed distinct immune-stromal landscapes, which appear to be associated with differential ERK pathway activity. Specifically, the hyperinflammatory subtype exhibited elevated expression of GSERK components, including DUSP1 and GADD45 family members, along with higher immune infiltration scores, suggesting a transcriptional state characterized by heightened ERK signaling. In contrast, the quiescent stromal subtype showed suppressed GSERK expression, lower immune cell signatures, and increased stromal features, indicative of a more immune-silent microenvironment. These findings are consistent with prior mechanistic studies showing that ERK signaling plays a critical role in modulating immune responses, including microglial activation, cytokine secretion, and endothelial dysfunction following cerebral ischemia. Moreover, ERK activity has been linked to stromal remodeling and astrocyte phenotypic transitions, processes that may underlie the divergent stromal profiles observed in our subtype analysis. While our current study does not establish a direct causal link between ERK activation and subtype specification, the correlative patterns strongly suggest that ERK signaling contributes to the functional polarization of the post-stroke microenvironment. Future studies integrating phosphoproteomics or single-cell perturbation approaches in stratified models will be essential to validate these regulatory relationships.

Our integration of multiple complementary machine-learning methods, like Boruta, SVM, LASSO, and random forest, provided rigorous confirmation of GADD45A, DUSP1, and GADD45B as robust biomarkers for ischemic stroke, substantially minimizing the risk of false discoveries inherent in genomic analyses (Zhou et al., 2023; Daneshvar and Mousa, 2023). Each algorithm, employing distinct statistical paradigms, independently underscored these GSERK candidates, thereby markedly enhancing our confidence in their biological relevance and predictive reliability (Kim et al., 2022). The methodological rigor demonstrated here exemplifies best practices in bioinformatics-driven biomarker discovery, where reproducibility across analytical frameworks is paramount to translational success (Shehab et al., 2022). To leverage these robust biomarker insights clinically, we developed a GSERK-based nomogram incorporating *GADD45A*, *DUSP1*, and *GADD45B* expression profiles into a composite predictive tool. This nomogram, featuring a strong bootstrap-validated C-index (0.72), demonstrated promising accuracy for predicting ischemic stroke risk, supporting its potential for enhancing clinical decision-making and patient stratification (Abdullah, 2024). DUSP1 emerged as a critical contributor with borderline statistical significance (OR = 2.57, *p* = 0.051) and consistently high predictive value, implicating its mechanistic role in ERK pathway regulation as central to ischemic injury and inflammation (Sun et al., 2021; Nunes-Xavier et al., 2019). Further prospective validation studies and carefully designed clinical trials are warranted to confirm the clinical utility, real-world applicability, and reliability of the nomogram as a precision medicine tool for ischemic stroke management (Patil et al., 2022).

The study has several notable limitations that must be acknowledged. First, although our analyses robustly identified GSERK genes as biomarkers and highlighted their mechanistic involvement through bioinformatic approaches, these findings remain theoretical in the absence of direct experimental validation using *in vivo* models or clinical patient samples. Second, potential clinical heterogeneity and confounding variables inherent to publicly available datasets may limit the direct translational applicability of our findings; thus, experimental validation in animal models or patient-derived biospecimens is warranted to confirm the biological functions and clinical relevance of the GSERK genes. Moreover, the molecular subgroups defined through immune-stromal clustering require further validation in larger and independent prospective cohorts to ascertain their reproducibility and prognostic value. Future research should prioritize the integration of multi-center clinical samples to validate both GSERK biomarkers and subgroup classifications, further strengthening their clinical utility. Finally, targeted therapeutic approaches modulating ERK signaling pathways merit rigorous exploration, particularly tailored to immune-stromal subgroup characteristics identified herein. These interventions may offer novel personalized strategies to mitigate inflammation-driven neuronal damage and facilitate reparative processes in ischemic stroke patients (Zheng et al., 2022; Qiu et al., 2022). While our study primarily focused on transcriptomic profiling and identified robust ERK pathway–associated gene signatures through multi-cohort integration and network analysis, we acknowledge that additional omics layers, such as epigenetic (e.g., DNA methylation) or proteomic data, could further enrich mechanistic insight. For example, examining whether the upregulation of DUSP1 is influenced by promoter demethylation or post-translational modifications may provide a deeper understanding of its regulation under ischemic conditions. Although such data were not available in the current datasets, future studies incorporating multi-omics approaches could offer a more comprehensive view of ERK pathway dysregulation in ischemic stroke. These integrative efforts may ultimately refine biomarker discovery and therapeutic targeting strategies.

From a drug development perspective, our analysis highlighted DUSP1 and GADD45B as promising ERK pathway–linked candidates. DUSP1, a dual-specificity phosphatase that negatively regulates MAPK activity, is known to attenuate inflammation and cellular stress responses. Pharmacological activation of DUSP1, or inhibition of upstream ERK signaling, could theoretically mitigate injury in hyperinflammatory stroke subtypes. Notably, several ERK inhibitors and MAPK pathway modulators (e.g., selumetinib, cobimetinib) are already in clinical use for other indications and may be repurposed pending further validation in stroke-specific models. Moreover, our nomogram incorporating GSERK markers offers a potential decision-support tool for individualized risk prediction and therapy stratification. As stroke treatment evolves beyond time-based protocols, molecular subtype–guided approaches could facilitate precision targeting of immune-modulatory therapies in both acute and subacute phases. Future work should integrate longitudinal data, clinical outcomes, and therapeutic responses to validate the predictive and actionable value of the proposed subtypes and biomarkers.

## Data Availability

The original contributions presented in the study are included in the article/supplementary material, further inquiries can be directed to the corresponding author.

## Footnotes

^1^ https://www.ncbi.nlm.nih.gov/geo/

^2^ https://www.genome.jp/kegg-bin/show_pathway?hsa04010

^3^ https://cn.string-db.org/
